# Supplementary material for: Large-scale crossbar arrays based on three-terminal MoS2 memtransistors
Source: Nat Commun. 2025 Oct 28;16:9518. doi: 10.1038/s41467-025-64536-2 (PMC12569200; doi:10.1038/s41467-025-64536-2)
Supplement: Supplementary file 1 — Supplementary Information [file 41467_2025_64536_MOESM1_ESM.pdf]

Supporting Information

Large-scale crossbar arrays based on three-terminal MoS_2 memtransistors

Thomas F. Schranghamer[1], Andrew Pannone[1], Jishnu M Kumar[1], Dev Krishna Thiyyadi Baiju[1], Chen Chen[2], Thomas McKnight[3,4], Sean Tadekawa[5], Evan Haines[5], Richard Ordonez[5], Cody Hayashi[5], Joan M. Redwing[2,3,4], and Saptarshi Das[1,2,4,6,]*

[1]Engineering Science and Mechanics, Penn State University, University Park, PA 16802, USA

[2]2D Crystal Consortium Materials Innovation Platform, Materials Research Institute, Penn State University, University Park, PA 16802, USA

[3]Materials Science and Engineering, Penn State University, University Park, PA 16802, USA

[4]Materials Research Institute, Penn State University, University Park, PA 16802, USA

[5]Naval Information Warfare Center Pacific, Pearl City, HI 96782, USA

[6]Electrical Engineering and Computer Science, Penn State University, University Park, PA 16802, USA

[*]Corresponding Author: Email address: sud70@psu.edu, das.sapt@gmail.com

Supplementary Note 1

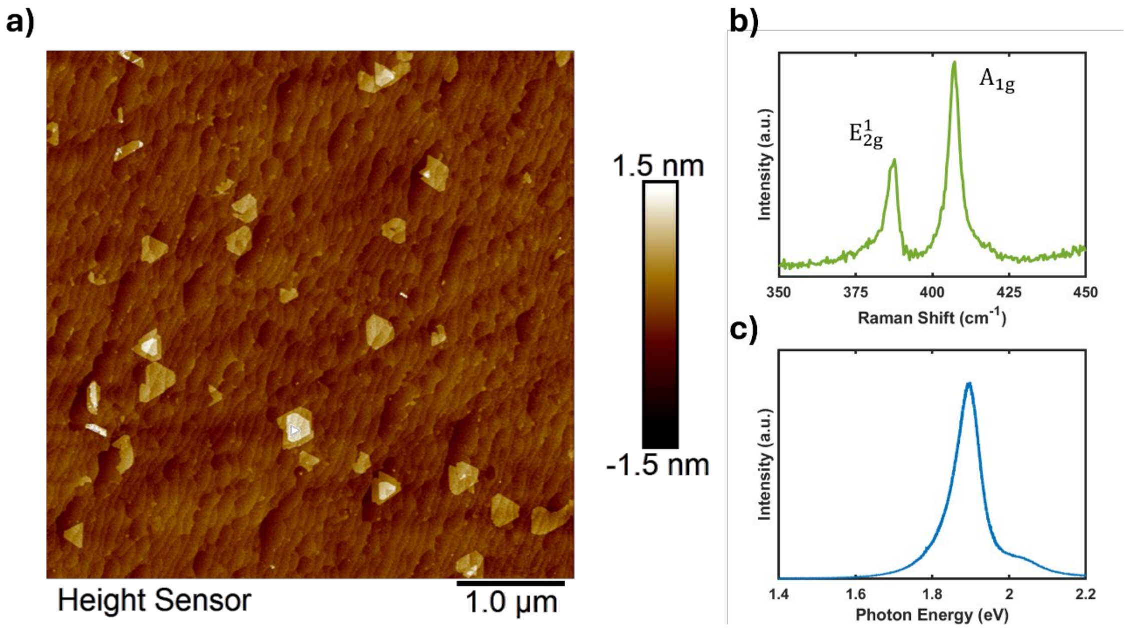

Supplementary Figure 1. Characterization of As-Grown MoS_2 Film. *a) Atomic force microscopy (AFM) of the as-grown MoS_2 film on sapphire, showing that the MoS_2 used in this study was predominantly monolayer with intermittent multilayer islands. b) Raman spectra of the as-grown MoS_2 film. The characteristic E_{2g}^{1} and A_{1g} peaks of MoS_2 can be clearly seen. c) Photoluminescence (PL) spectra of the as-grown MoS_2 film showing an A-exciton peak position of ~1.9 eV, which is typical for as-grown monolayer MoS_2 on sapphire.*

Supplementary Note 2

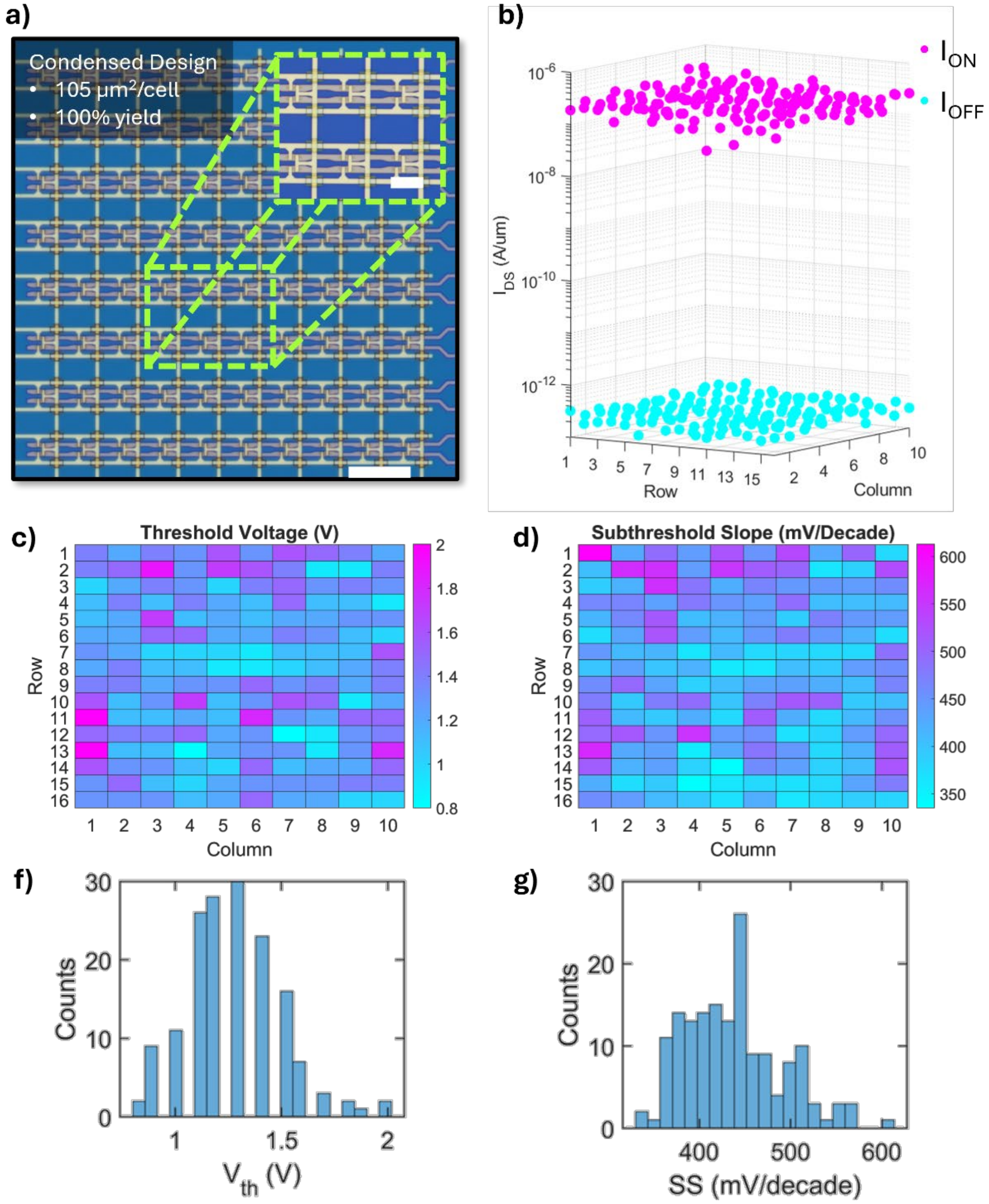

Supplementary Figure 2. Overview of Condensed Crossbar Array Architecture. *a) Optical image of a representative 16×10 MoS_2-memtransistor-based crossbar array based on an alternative design from that reported in* ***Figure 1****, denoted here as 'Condensed'. This design features a non-volatile memory (NVM) cell area of 105 μm^2/cell, an ~84.5% reduction from the base design discussed in* ***Figure 1*** *and elsewhere in the main text; this corresponds to an information density of ~0.95 Mb/cm^2 for 1-bit operation. Inset shows a zoomed-in image of constituent memtransistors. Scale bar denotes 25 μm (10 μm for inset). b) Three-dimensional scatter plot showing distribution of ON-state and OFF-state currents taken at a drain-to-source voltage (V_{DS}) of 1 V, denoted as I_{ON} (pink) and I_{OFF} (cyan), respectively, across the 16×10 array. Notably, all 160 devices in the array were found to work (100% yield) despite the reduction in cell area from the base design. c-d) Maps of threshold voltage (V_{th}) and subthreshold slope (SS), respectively, across the array. e-f) Histograms of V_{th} and SS, respectively, for the 160 devices in the array.*

Supplementary Note 3

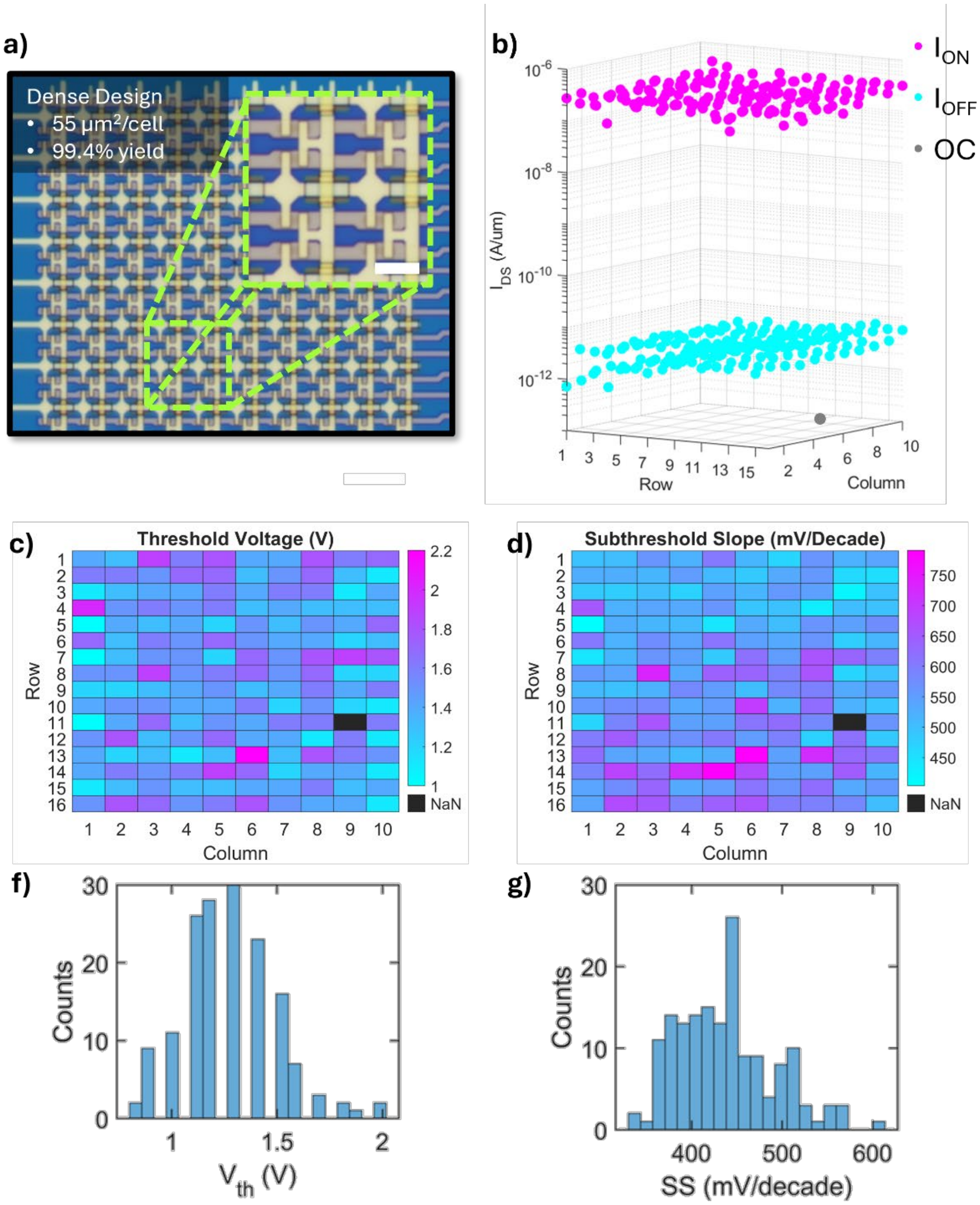

Supplementary Figure 3. Overview of Dense Crossbar Array Architecture. *a) Optical image of a representative 16×10 MoS_2-memtransistor-based crossbar array based on an alternative design from that reported in* ***Figure 1****, denoted here as 'Dense'. This design features an NVM cell area of 51.5 μm²/cell, an ~92.4% reduction from the base design discussed in* ***Figure 1*** *and elsewhere in the main text; this corresponds to an information density of ~1.94 Mb/cm² for 1-bit operation. Inset shows a zoomed-in image of constituent memtransistors. Scale bar denotes 20 μm (5 μm for inset). b) Three-dimensional scatter plot showing distribution of I_{ON} (pink) and I_{OFF} (cyan) taken at V_{DS} = 1 V across the 16×10 array; devices/cells marked in gray registered as an open circuit (OC) when measured. Notably, 159/160 devices in the array were found to work (99.4% yield) which is comparable to the base design despite the reduction in cell area. c-d) Maps of V_{th} and SS, respectively, across the array. e-f) Histograms of V_{th} and SS, respectively, for the 159 working devices in the array.*

Supplementary Note 4

Table 1: Crossbar-Level Demonstrations based on 2D Materials

Device Structure	Active Length/Width	Cell Area	Yield (%)	Array Size	Array Configuration	Switching Energy Consumption	Retention (Exp./Calc.)	ON/OFF Ratio	Terminals	Multi-level Capability?	Reference
MoS_2 Memtransistor	1 um / 1 um	676 um^2	92.2	64×32	1T	~ 20 pJ (base), ~ 0.2 fJ (peak)	> $4*10^3$ s / ~3.17 yrs	~10^5	3	Y	This Work
MoS_2 Memtransistor	1 um / 1 um	~ 105 um^2	100	16×10	1T	~ 20 pJ (base), ~ 0.2 fJ (peak)	> $4*10^3$ s / ~3.17 yrs	~10^5	3	Y	This Work
MoS_2 Memtransistor	1 um / 1 um	~ 51.5 um^2	99.4	16×10	1T	~ 20 pJ (base), ~ 0.2 fJ (peak)	> $4*10^3$ s / ~3.17 yrs	~10^4	3	Y	This Work
MoS_2 FGFET	~3.1 um / ~49.5 um	~ 32500 um^2	83.1	32×32	1T	NA (~ 8 - 20 nJ estimated)	> $3.5*10^3$ s / NA	~ 100 - 300	3	Y	[1]
MoS_2 Memtransistor	0.4 um / 20 um	~ 4000 um^2	64	10×10	1T	~ 20 fJ	> 800 min / NA	> 100	3	Y	[2]
MoS_2 Memtransistor	0.9 um / 0.7 um	~ 5 um^2	NA	10×9	1T	~ 20 nJ	> 10^5 s / > 10 yrs	> 1000	4	Y	[3]
MoS_2 Memristor	1 um / 50 um	~ 7250 um^2	NA	2x2	1T-1R	NA	> $3.6*10^3$ s / NA	~ 10^5 - 10^6	2	Y	[4]
Au/h-BN/Au Memristor	5 um / 5 um	~ 100 um^2	NA	10×10	1R	5.47 pJ	NA / NA	~10^6	2	Y	[5]
Au/MoS_2/Ag Memristor	30 um^2	NA	NA	4x4	1R	NA	> $2*10^5$ s / NA	~10^5	2	N	[6]
Pt/MoS_2/Ag Memristor	10 um / 10 um	NA	NA	4x4	1R	20 uJ	> 10^4 s / NA	~ 10	2	N	[7]
Pt/MoS_2/Ti Memristor	5 um / 5 um	~ 900 um^2	NA	6x6	1R	~ 40 pJ	> 10^5 s/ > 3 yrs	~ 100	2	Y	[8]
Au/h-BN/Au Memristor	3 um / 3 um	~25 um^2	98 (DA)	10×10	1R	~ 2.9 uJ	> 100 s / NA	> 10^5	2	Y	[9]

NA: Not announced

DA: Different arrays; data taken across multiple experimental crossbar arrays

Supplementary Table 1. Crossbar-Level Demonstrations based on 2D Materials. *Benchmarking of this work against extant demonstrations of crossbar arrays based on 2D materials[1-9]. The cell areas for the arrays developed in this work range from 676 μm^2 (base design) to 51.5 μm^2 (densest design), demonstrating our ability to successfully scale our crossbar array architectures to information densities of up to 1.94 Mb/cm^2 (assuming 1-bit operation). However, even the base design displays a significantly higher information density than almost all experimentally demonstrated 2D-material-based arrays to-date; while some reports have demonstrated cell areas down to ~5 μm^2 per cell, the ultimate array size of those works was significantly less than that achieved in this effort. Notably, only a single other work has demonstrated comparable array sizes to ours, though at a significantly lower yield than what we have thus far demonstrated. Our array architectures also compare favorably in terms of switching energy, ranging from tens of pJ (base) to below 1 fJ (peak); all switching energies shown here were estimated using the equation Energy = Time × Current × Voltage, which is commonly used to estimate switching energy for NVMs. The terms "base" and "peak" included in the assessment of our work refer to the pulse time (switching time), with base referring to our typical pulse time of 100 ms and peak referring to our minimum confirmed pulse time of 1 µs. While several of the other works included in this comparison also utilized ultrafast switching times, our NVM capabilities being controlled by the gate means that the switching current of our devices is limited to the gate leakage current, which remained in the region of several tens of pA even at the largest gate biases applied (~10 V), thus allowing for severely reduced energy expenditure. Other metrics (retention, ON/OFF ratio, number of terminals, and ability to realize multiple memory states) compare similarly to other demonstrations, indicating suitability for future applications.*

2D memtransistors such as those explored in this work offer several compelling advantages over conventional neuromorphic memory technologies such as resistive random-access memory (RRAM), phase-change memory (PCM), and ferroelectric memories. Conventional two-terminal RRAM devices frequently encounter challenges related to variability[24] and sneak path currents, typically requiring access/selector devices in each NVM cell that increase complexity and footprint[2, 3, 24-26]. Three-terminal memtransistors address these issues with electrostatic gating, providing lower leakage currents and more linear conductance modulation[2, 3]. While RRAM technology is relatively mature and can be densely integrated, 2D memtransistor arrays achieve comparable density with the added benefit of atomically-thin channels that could enable 3D stacking of layers[27-29]. An overview of the NVM cell areas achieved in various MoS_2-based crossbar array demonstrations is presented as part of the benchmarking in **Supplemental Table 1**. However, a major trade-off is that MoS_2 devices are at an earlier stage; improving their endurance and uniform wafer-scale fabrication are ongoing research challenges that are beyond the scope of this study. PCM devices, while relatively stable and mature, rely on thermally induced phase changes that lead to higher energy consumption and slower speeds compared to the charge-trapping mechanisms in MoS_2 devices[24]. Notably, PCMs have been demonstrably integrated into memory chips, though adding them onto logic circuits is non-trivial due to thermal budget constraints[30]. In contrast, MoS_2 devices are relatively flexible: they can be fabricated at back-end compatible conditions or transferred onto substrates, potentially allowing monolithic integration of sensing, memory, and logic on the same chip[27, 28]. Ferroelectric memories, though CMOS-compatible and fast-switching, often exhibit abrupt polarization transitions, complicating analog weight tuning[31]. MoS_2 memtransistors, by contrast, inherently offer more linear, gradual, and energy-efficient analog conductance updates[2, 3, 32-35].

It should also be noted that numerous works have investigated hybrid memory architectures (e.g., an MoS_2 channel FET with a ferroelectric gate material[36-38]) to harness the strengths of each. These technologies, while promising, use different material stacks and operational principles from those

explored in this work, and as such represent a complementary rather than directly comparable approach. Overall, while each memory technology has distinct advantages, we believe the combination of non-destructive operation, runtime tunability, and integration flexibility offered by our MoS_2 memtransistor crossbar arrays provides a compelling path for future low-power, adaptable neuromorphic systems.

Supplementary Note 5

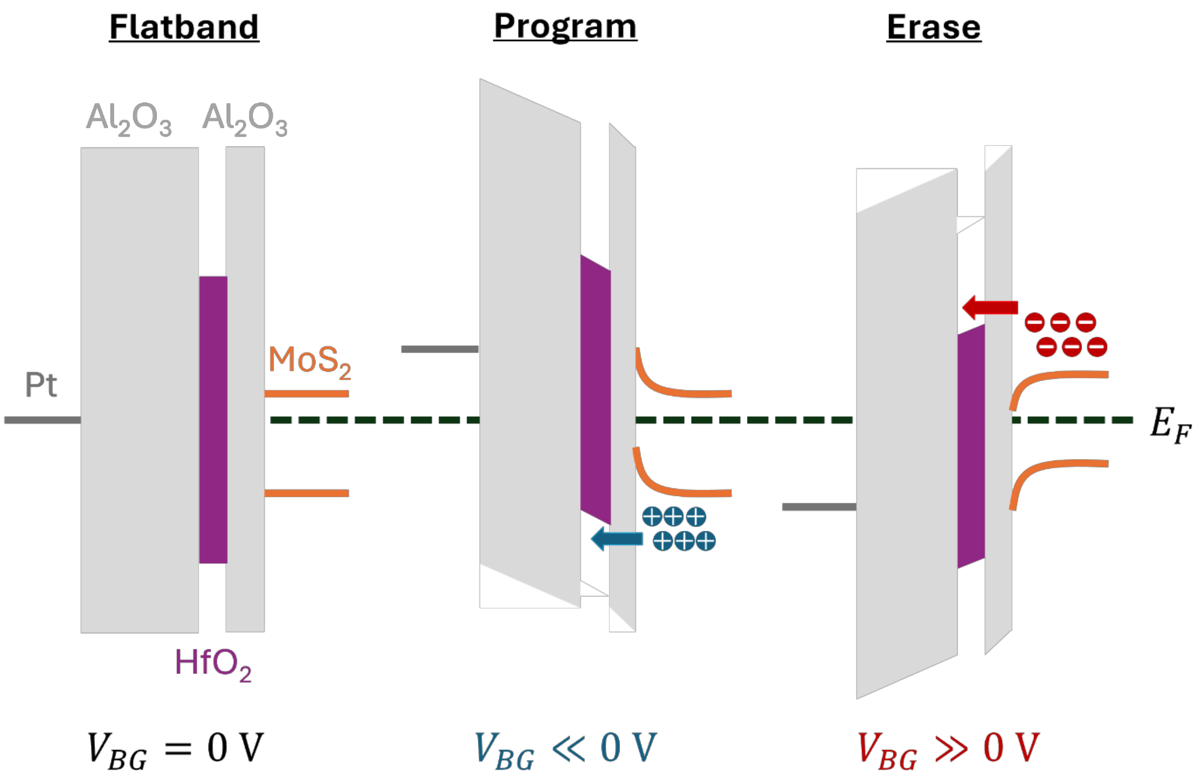

Supplementary Figure 4. Band diagram of $Al_2O_3/HfO_2/Al_2O_3$ gate dielectric stack. *The $Al_2O_3/HfO_2/Al_2O_3$ gate dielectric stack enables non-volatile memory in 2D MoS_2 memtransistors by allowing the trapping/detrapping of charge carriers in the HfO_2 (charge-trapping) layer when bias pulses of sufficient magnitude are applied to the back-gate. The polarity of the pulse determines which charge carriers are trapped/detrapped, with holes (electrons) being trapped when negative (positive) pulses are applied, and vice versa. These trapped charges screen the electric field across the MoS_2 channel, changing the conductance of the device and allowing for the realization of distinct conductance (memory) states.*

Supplementary Note 6

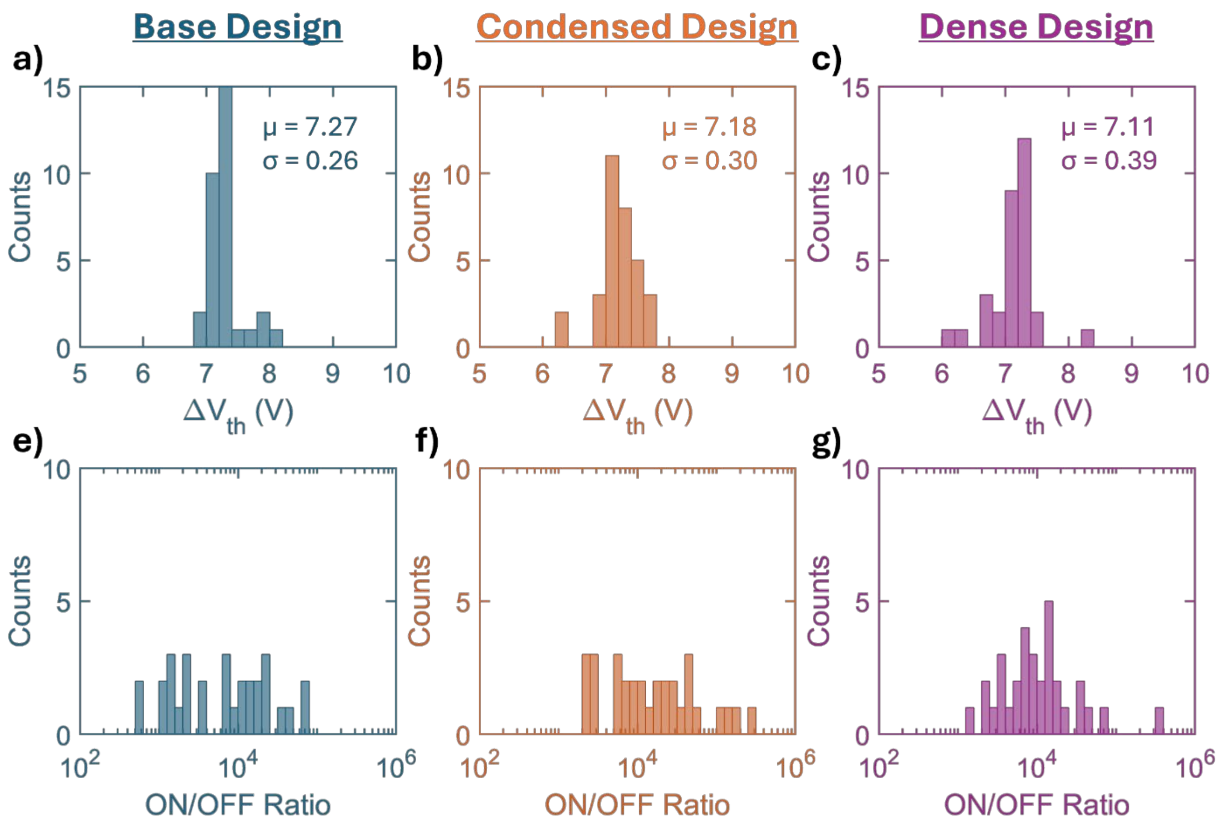

Supplementary Figure 5. Analysis of Memory Window and Read Margin in MoS_2-Memtransistor-based Crossbar Arrays. *a-c) Histograms of the memory window (ΔV_{th}) for cells in crossbar arrays of the base design (**Figure 1**), condensed design (**Supplementary Information 2**), and dense design (**Supplementary Information 3**), respectively. The means (μ) and standard deviations (σ) are noted for each case. d-f) Histograms of the read margin (ON/OFF ratio) for same arrays as in (a-c). Similar distributions, means, and standard deviations can be seen for all cases, indicating that the different crossbar array architectures developed in this work operate similarly despite design variations.*

Supplementary Note 7

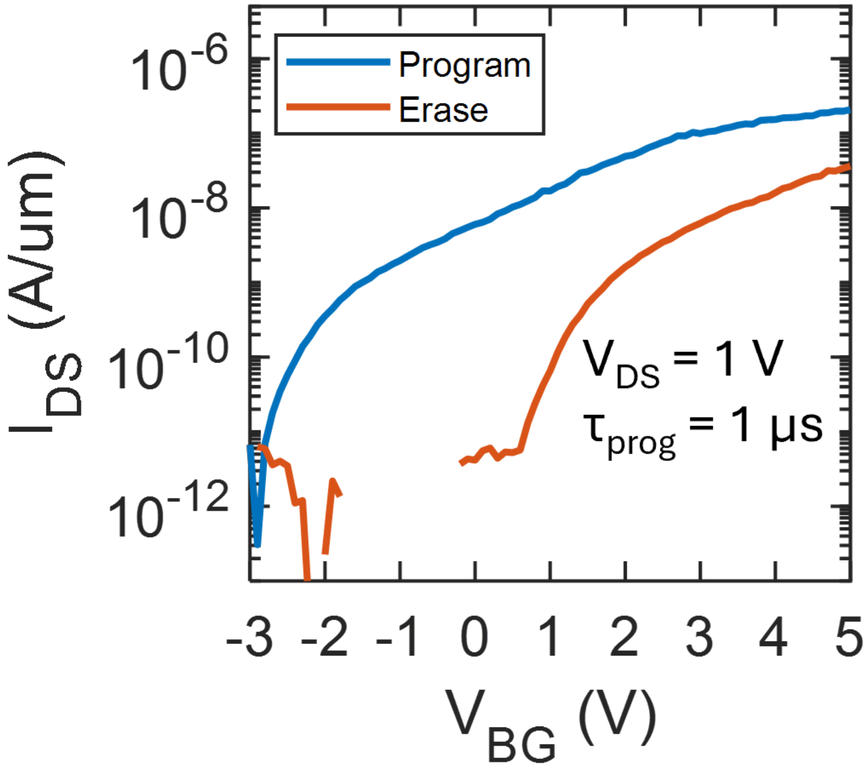

Supplementary Figure 6. 1 μs Program/Erase Pulse Demonstration. *Transfer characteristics, i.e., drain-to-source current (I_{DS}) versus back-gate voltage (V_{BG}), taken at V_{DS} = 1 V of a representative memtransistor before and after application of a 1 μs programming pulse (-10 V). As can be clearly seen, the pulse shifts the V_{th} of the device, indicating a shift to a different distinct conductance state (weight). This establishes that 1 μs pulse times can program/erase the constituent NVM cells of the crossbar arrays developed and investigated in this work, thus indicating that the arrays may be operated at high speeds (frequencies) than utilized in this work.*

Supplementary Note 8

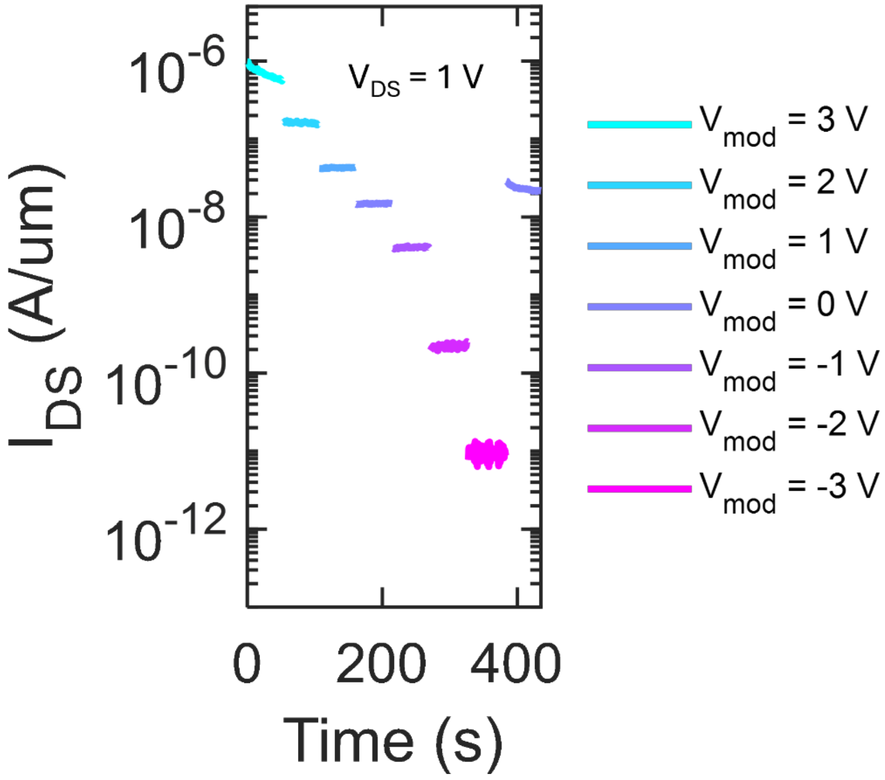

Supplementary Figure 7. Demonstration of In-State Conductance/Weight Modulation. *I_{DS} monitored over time for a representative memtransistor programmed into the ON-state through the application of a 100 ms, -10 V back-gate bias pulse and subsequently read at different modulatory back-gate biases (V_{mod}) for a constant V_{DS} = 1 V. Note that, despite the device being programmed to a set conductance state through the aforementioned programming event, varying V_{mod} allows for the effective realization of multiple in-state current/conductance levels, thus presenting an avenue for the dynamic potentiation/depression of weights in crossbar arrays through the application of a positive/negative V_{mod} to the respective gate lines. Also note that, for the case of V_{mod} = 0 V, the same current/conductance level is retained when V_{mod} switches between values, indicating that this modulatory process does not affect the programmed device state.*

Supplementary Note 9

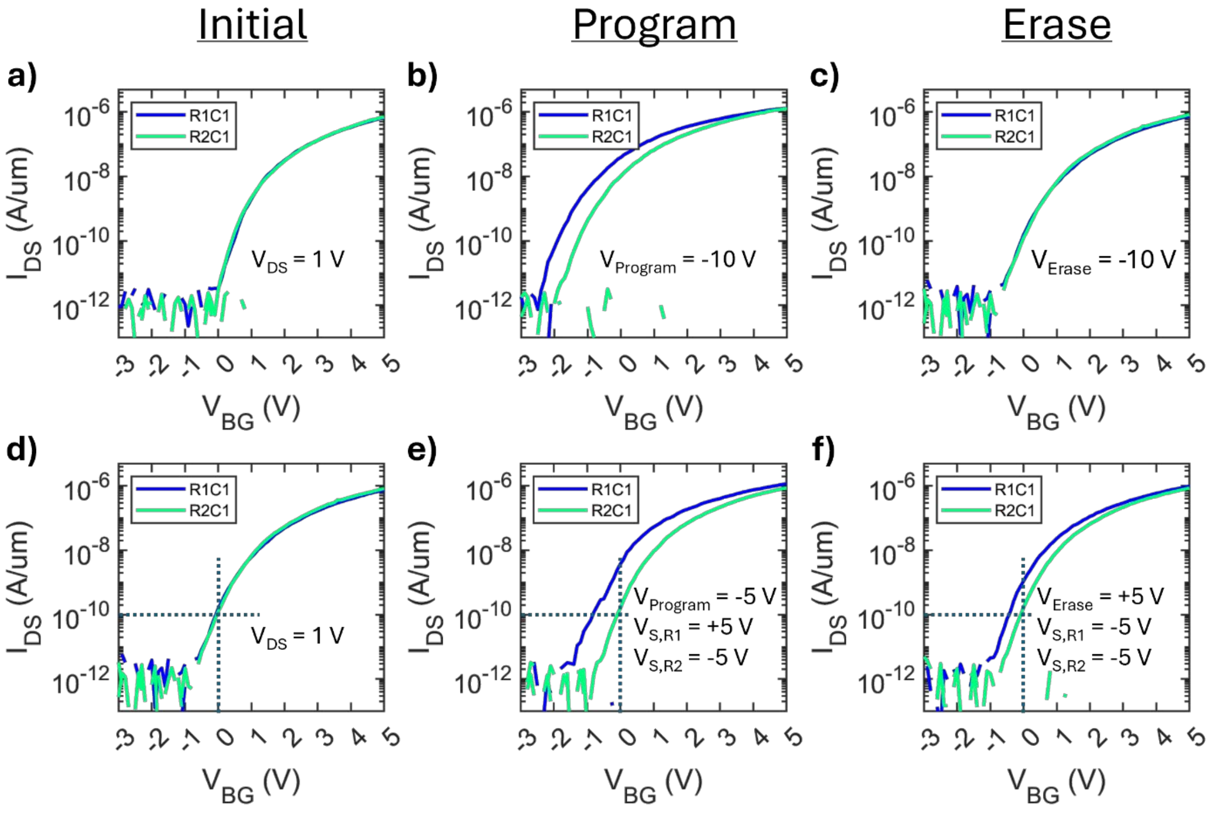

Supplementary Figure 8. Bias Scheme Testing. *(a-c) Testing of two devices (R1C1 and R2C1) on the same word/gate-line of a representative MoS_2-memtransistor-based crossbar array using a full biasing scheme. (a) The as-fabricated devices are read before being (b) programmed to high conductance states using a negative voltage pulse ($V_{Program}$ = -10 V) and (c) erased to their initial conductance states using a positive voltage pulse (V_{Erase} = +10 V). All other access lines are held at 0 V during pulsing. (d-f) Testing of R1C1 and R1C2 using a half-biasing scheme. (d) The devices are read before being (e) subjected to a negative gate voltage pulse ($V_{Program}$ = -5 V) while the source of Row 1 ($V_{S,R1}$) is held at +5 V and the source of Row 2 ($V_{S,R2}$) is held at -5 V. This maximizes the gate-to-source voltage (VGS) across R1C1 while minimizing the VGS across R2C1; as a result, only R1C1 experiences a change in its conductance state. When the biases are then flipped (f), R1C1 is again the only device to display any change, returning closer to its initial conductance state.*

Supplementary Note 10

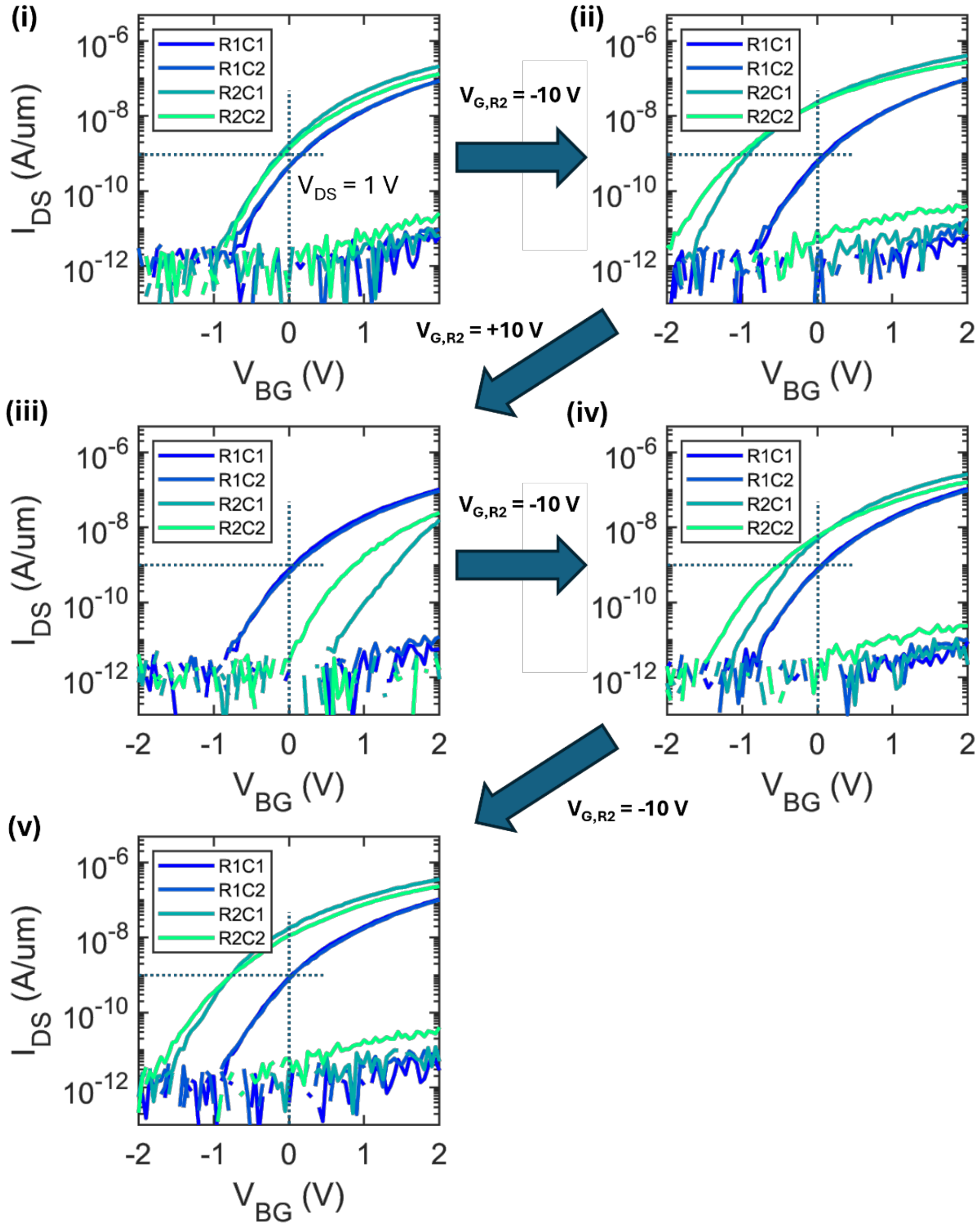

Supplementary Figure 9. Word/Gate-Line Isolation Testing. *(i-v) Demonstration of gate line isolation in a representative 2×2 crossbar array. Dotted lines are to help show shift in transfer characteristics after each program/erase operation. All four as-fabricated devices in the 2×2 array (R1C1, R1C2, R2C1, and R2C2) were read (i) before the word/gate-line in Row 2 (R2) was sequentially subjected to (ii) a -10 V bias pulse ($V_{G,R2}$), (iii) a +10 V bias pulse, (iv) a -10 V bias pulse and (v) a -10 V bias pulse, for 100 ms each. As can be seen, while the devices in R2 (R2C1 and R2C2) were programmed and erased as expected when exposed to negative and positive bias pulses, respectively, the devices in R1 (R1C1 and R1C2) did not show any appreciable shift in their transfer characteristics throughout all applied pulses, confirming the isolation of the separate word/gate lines.*

Supplementary Note 11

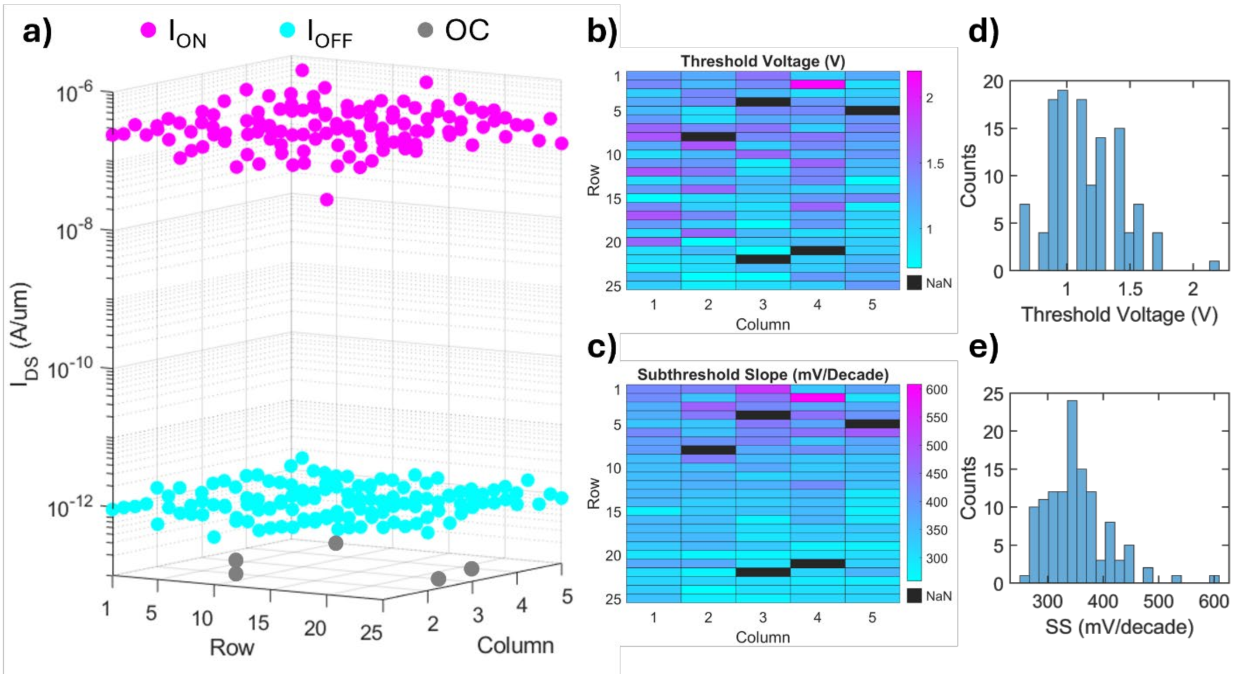

Supplementary Figure 10. Overview of 25×5 Sub-Array. *a) Three-dimensional scatter plot showing distribution of I_{ON} (pink) and I_{OFF} (cyan) taken at V_{DS} = 1 V across the 25×5 sub-array shown and discussed in* ***Figure 2;*** *devices/cells marked in gray registered as an open circuit (OC) when measured. 120/125 devices in the array were found to work (95% yield). b-c) Maps of threshold voltage (V_{th}) and subthreshold slope (SS), respectively, across the array. Nonworking devices are marked as NaN. d-e) Histograms of V_{th} and SS, respectively, for the 120 working devices in the array.*

Supplementary Note 12

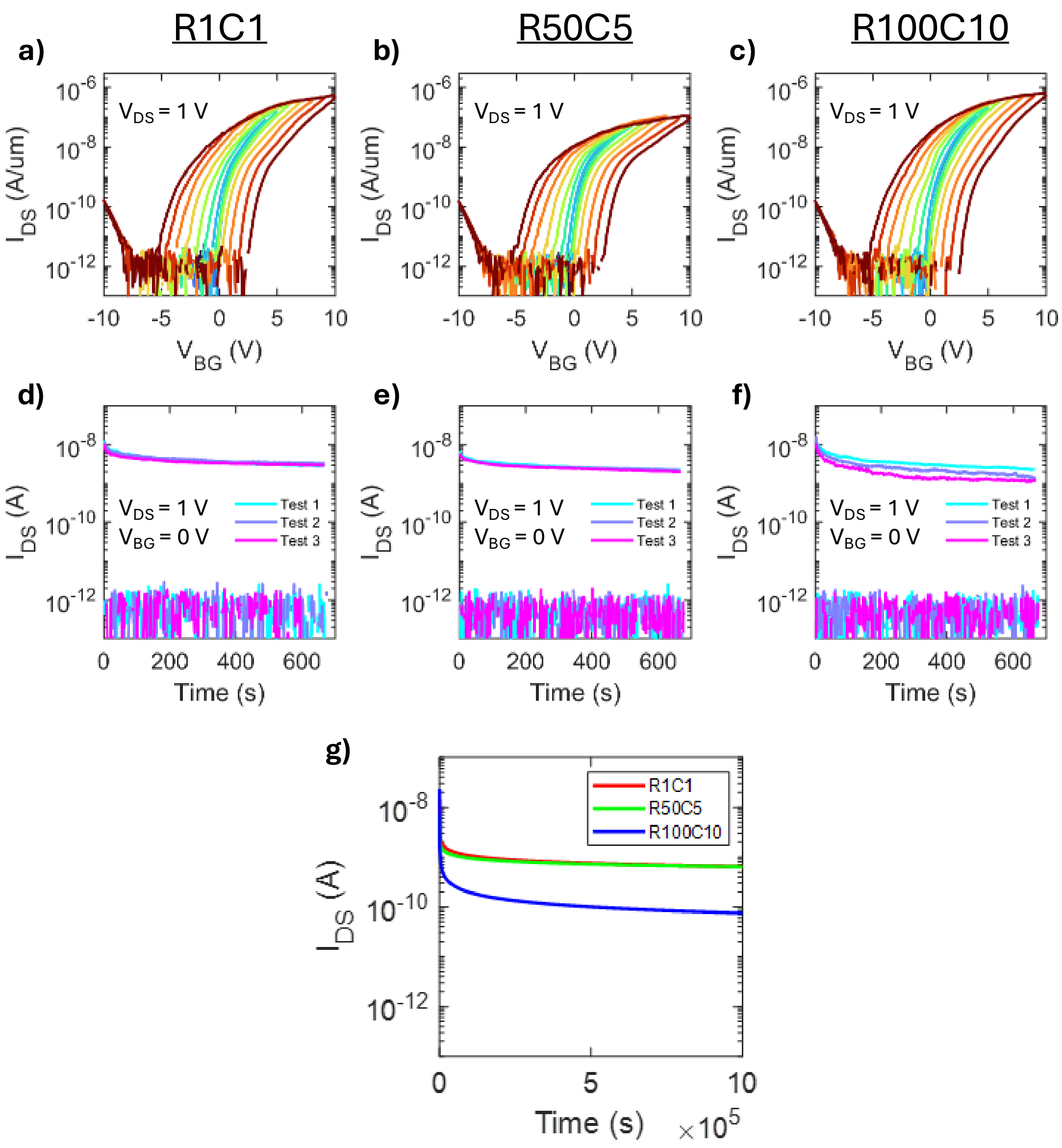

Supplementary Figure 11. Memory Testing across 100×10 Crossbar Array. *a-c) Hysteresis loops for devices located at the top (R1C1), middle (R50C5), and bottom (R100C10) of a representative 100×10 array. For each device, multiple hysteresis loops were taken by sweeping the back-gate voltage between +/- 2 V, +/- 3 V, +/- 4 V, +/- 5 V, +/- 6 V, +/- 7 V, +/- 8 V, +/- 9 V, and +/- 10 V so as to determine the presence/size of the memory window for different program/erase voltages; a sizable memory window of ~10 V can be noted for +/- 10 V sweeps irrespective of array position, indicating that array size has minimal effect on program/erase capabilities. d-f) Retention tests for the top, middle, and bottom devices shown in (a-c), respectively. Devices were subjected to a -10 V programming pulse (putting them in the ON-state) and a +10 V erasing pulse (putting them in the OFF-state) and read at a gate voltage of 0 V and drain voltage of 1 V for ~600 seconds (10 minutes) to observe the decay in their ON/OFF ratio over time. This test was conducted three times to observe retention/programming consistency. The timing of each program/erase bias pulse was 100 ms. g) To analyze the long-term retention and uniformity of these devices, a simple power law fit was extracted for the median retention curve of each device and plotted over 10^6 seconds (~11.6 days). The final fitted ON-current values are all >100 pA; if the OFF-current for each device remains constant at ~2 pA, the ON/OFF ratio should remain > 100 for over a week, which is more than sufficient for edge computing applications.*

Supplementary Note 13

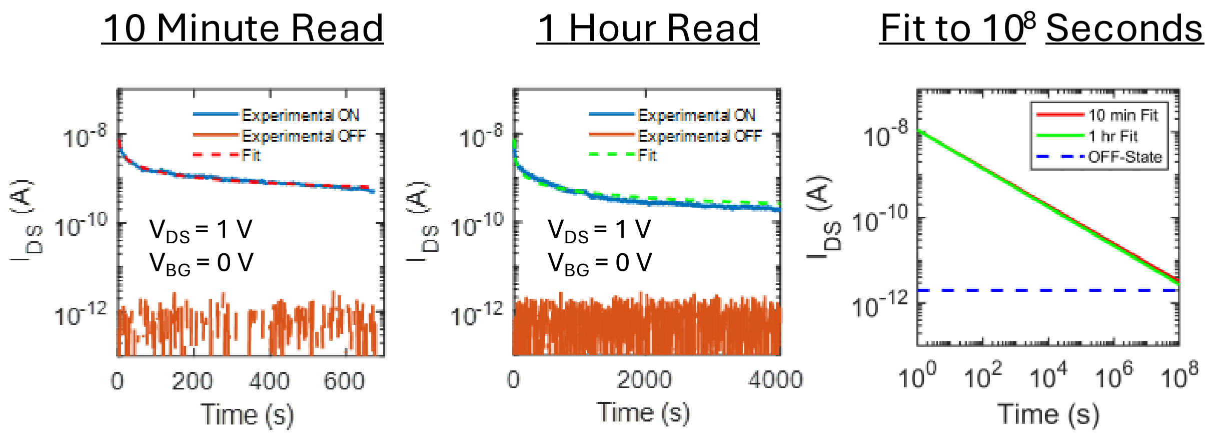

Supplementary Figure 12. Long Term Memory Testing. *To verify the accuracy of the fits shown in* ***Supplemental Note 12****, the ON-state and OFF-state of a representative device/cell were read over periods of ~10 minutes (left column) and ~1 hour (middle column) and simple power law fits were calculated. These fits were then plotted (right column) over* 10^8 *seconds (~3.5 years), during which time they remained above the designated OFF-state current (~2 pA); notably, the 10 minute and 1-hour fits were in close agreement throughout the entire time span, indicating that the 10 minutes fit discussed above are relatively accurate. Additionally, no degradation in the OFF-state was noted even for the longer retention tests, indicating that the change in ON/OFF ratio over time will predominantly depend on the change in ON-state conductance.*

Supplementary Note 14

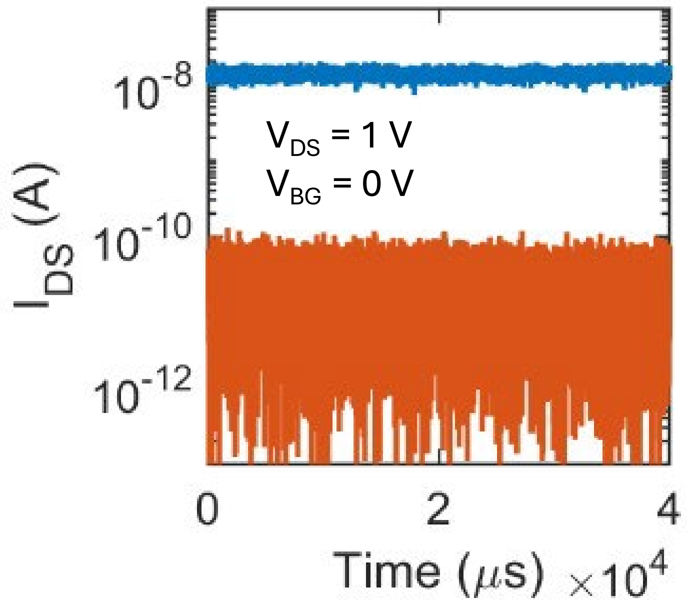

Supplementary Figure 13. Minimum Write/Read Stability Testing. *To verify memory stability even at the minimum confirmed write/read times of 1 μs and 4 μs, respectively, the ON-state (blue) and OFF-state (orange) of a representative device/cell were read over a period of 40 ms using ten-thousand 4 μs read pulses. Programming/erasing were each achieved using a single +/- 10 V write pulse, respectively, with a 1 μs pulse time. A read margin >10^2 is maintained throughout the entire timespan, indicating that the charge-trapping memory effect remains non-volatile even at low write energies. Note that the high frequency read setup used for this experiment possesses a noise floor of ~10^{-10} A; as a result, the actual OFF-state current may be closer to that presented in* ***Supplementary Figures 11-12*** *with a correspondingly larger read margin.*

Supplementary Note 15

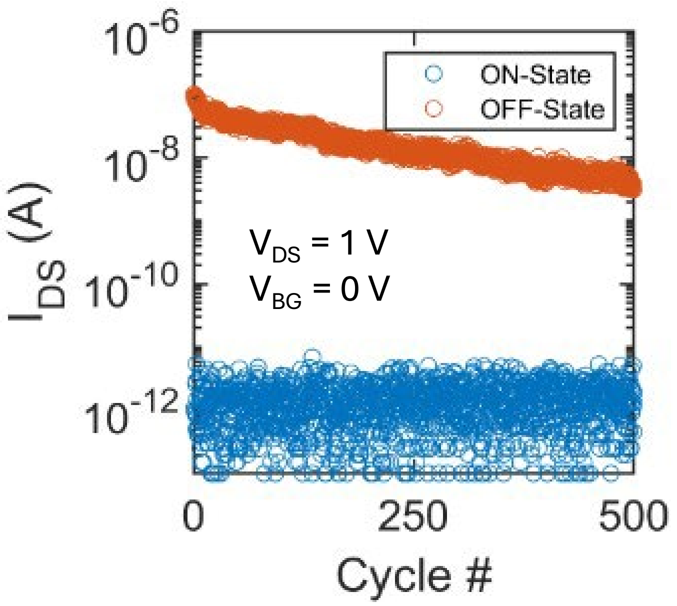

Supplementary Figure 14. Memtransistor Endurance Testing. *ON-state and OFF-state of a representative device/cell taken over 500 program/erase cycles ($V_{program}$ = -10 V, V_{erase} = +10 V, t_{pulse} = 100 ms). Some degradation in the memory ratio between the ON-state and the OFF-state was seen during cycling; however, the read margin remained >10^2, indicating suitable endurance for NVM cell application.*

Supplementary Note 16

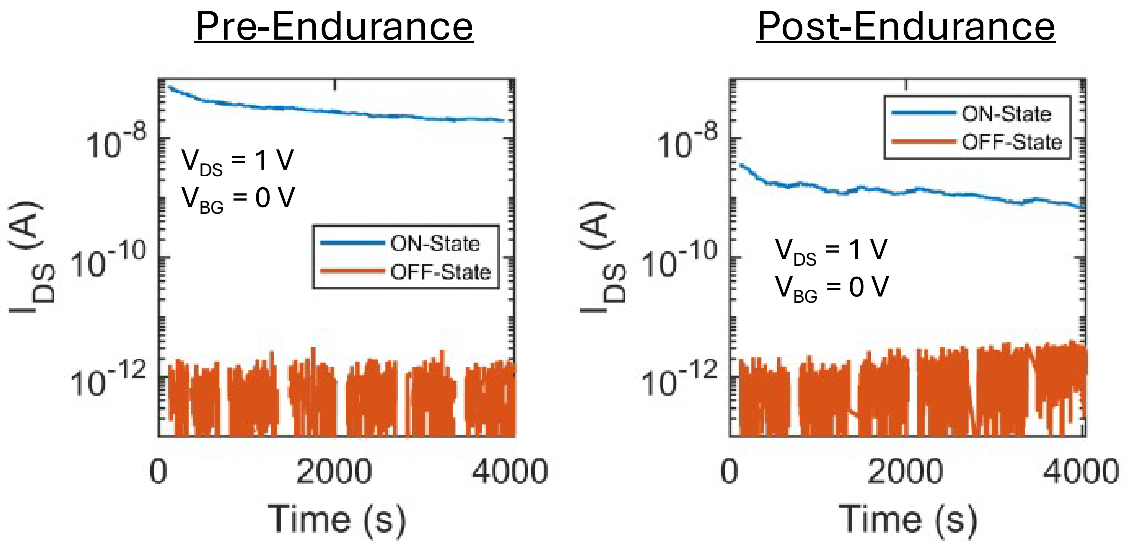

Supplementary Figure 15. Pre-/Post-Endurance Retention Testing. *ON-state and OFF-state of a representative device/cell taken over ~1 hour before (left) and after (right) the endurance testing shown in* ***Supplementary Figure 14****. For both cases,* $V_{program}$ *= -10 V,* V_{erase} *= +10 V, and* t_{pulse} *= 100 ms. Some degradation in the read margin between the ON-state and the OFF-state was seen after cycling; however, the read margin remained >102 with little degradation in long-term retention, indicating minimal write disturbance from repeated program/erase cycles.*

Degradation in the read margin following endurance testing may stem from several physical mechanisms. For instance, the relatively high defect density of Al_2O_3, while beneficial for phenomena such as trap-assisted tunneling, can lead to a gradual accumulation of charge carriers in trap states over the course of many program/erase cycles, shifting the endurance characteristics[39]. Additionally, high electric field stresses have previously been shown to induce irreversible charge trapping in HfO_2-related defects, permanently damaging the charge-trapping gate stack[40]. This may be addressed in future work by doping the HfO_2 charge-trapping layer with Al, reducing the concentration of oxygen vacancies and introducing reversible deep trap states that can reliably filled/depleted during memory operations to help achieve distinct conductance states[41]. Rapid thermal annealing under in an oxygen environment has also been shown to heal oxygen vacancies in similar charge-trapping stacks, improving their resistance to degradation under high electric fields (i.e., endurance)[42].

Supplementary Note 17

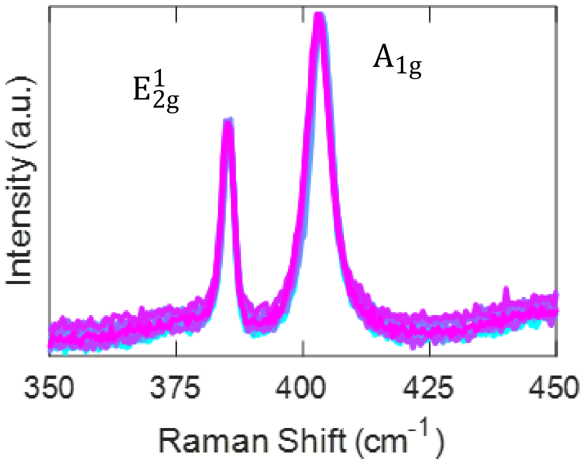

Supplementary Figure 16. Raman Spectroscopy Analysis of 64×32 Crossbar Array. *The Raman spectra was taken across nine points corresponding to the corners, sides, and center of the array with a 532 nm laser. The mean E^1_{2g} (in-plane) and A_{1g} (out-of-plane) peak locations were found to be 385.26 cm^{-1} and 403.15 cm^{-1}, respectively, with a mean peak separation of 17.89 cm^{-1}.*

Supplementary Note 18

Table 2: Comparison to Neural Network Accelerators[1]

Designation	Exp./Sim	Type	Process (nm)	Activation Resolution	Weight Resolution	Clock Speed	Weight Storage	Array Size	Throughput (TOPS)	Density (TOPS/mm^2)	Reference
MoS_2-Memtransistor-based Accelerator	Exp.	Memtransistor	Custom	1 bit	1 bit	10 Hz (base)<br>0.250 MHz (peak)<br>10 MHz (projected)	Charge-trapping	64×30<br>(Sub-Array)	$1.23*10^{-7}$ (base)<br>$3.06*10^{-3}$ (peak)<br>1.23 (projected)	$9.47*10^{-7}$ (base)[2]<br>$2.37*10^{-3}$ (peak)[2]<br>0.95 (projected)[2]	This Work
Condensed Memtransistor Accelerator (Proj.)	Exp.	Memtransistor	Custom	1 bit	1 bit	10 MHz	Charge-trapping	64×32	1.31	6.10 (array-only)	Projection[3]
Dense Memtransistor Accelerator (Proj.)	Exp.	Memtransistor	Custom	1 bit	1 bit	10 MHz	Charge-trapping	64×32	1.31	12.4 (array-only)	Projection[3]
NVIDIA T4	Exp.	Full-CMOS	12	8-bit int	8-bit int	2.6 GHz	NA	NA	22.2, 130 (peak)	0.04, 0.24 (peak)	[10]
Google TPU v1	Exp.	Full-CMOS	28	8-bit int	8-bit int	700 MHz	NA	NA	21.4, 92 (peak)	0.06, 0.28 (peak)	[11]
Habana Goya HL-1000	Exp.	Full-CMOS	16	16-bit int	16-bit int	2.1 GHz (CPU)	NA	NA	63.1	NA	[12]
DaDianNao	Sim.	Full-CMOS	28	16-bit fixed-pt.	16-bit fixed-pt.	606 MHz	NA	NA	5.58	0.08	[13]
UNPU	Exp.	Full-CMOS	65	16 bits	1 bit	200 MHz	NA	NA	7.37	0.46	[14]
Reference mixed-signal	Exp.	Full-CMOS	28	1 bit	1 bit	10 MHz	NA	NA	0.478	0.1	[15]
ISAAC	Exp.	RRAM-CMOS	32	16 bits	16 bits	1.2 GHz	ReRAM	128×128	41.3	0.48	[16]
Newton	Exp.	RRAM-CMOS	32	16 bits	16 bits	1.2 GHz	ReRAM	128×128	NA	0.68	[17]
PUMA	Exp.	RRAM-CMOS	32	16 bits	16 bits	1.0 GHz	ReRAM	128×128	26.2	0.29	[18]
PRIME	Sim.	RRAM-CMOS	65	6 bits	8 bits	3.0 GHz (CPU)	ReRAM	256×256	NA	NA	[19]
Memristive Boltzmann machine	Sim.	RRAM-CMOS	22	32 bits	32 bits	3.2 GHz (CPU)	ReRAM	512×512	NA	NA	[20]
3D-aCortex	Exp.	RRAM-CMOS	55	4 bits	4 bits	1.0 GHz	NAND flash	64×128	10.7	0.58	[21]
Analog-AI Using Dense 2D Mesh	Sim.	RRAM-CMOS	14	8 bits	Analog	1.0 GHz	PCM	512×512	376.7	NA	[22]

[1]: Adapted from ref. 10, 23 [2]: Array-only estimation [3]: Projection based on current experimental crossbar architectures
NA: Not announced

Supplementary Table 2. Benchmarking Against Emerging Neural Network Accelerators. *Benchmarking of this work against extant demonstrations of large-scale neural network accelerators[10-23]. We compare against the MNIST inference testing (i.e., digit classification) performed on a 64×30 subsection of the 64×32 (2 kb) array discussed in the main text, though we also include projections for full-scale operations on accelerators using the alternative crossbar architectures detailed in* ***Supplemental Note 2-3****. The activation and weight resolution are listed as 1 bit (ON/OFF) for our logic accelerators despite the analog nature of our NVMs due to binary images being used for this investigation. For all cases, clock speed is based on the time required for logic operations to be conducted (i.e., read operations when inputs are applied). For our accelerators, base refers to our typical read time of 100 ms (10 Hz), peak refers to our minimum confirmed read time of 4 μs (0.25 MHz), and projected refers to a theoretical clock speed as permitted by the final system (10 MHz). For assessing the throughput of our 64×10 sub-array, the number of columns being utilized at a time was restricted to 1 due to experimental limitations in assessing outputs in parallel; the listed projections are for fully parallelized array operations. A base throughput of $1.23×10^{-7}$ trillion-operations-per-second (TOPS), a peak throughput of $3.06×10^{-3}$ TOPS, and a projected throughput of 1.23 TOPS were estimated for our experimental 64×10 demonstration; this increases to 1.31 TOPS for our projected 64×32 accelerators. Another key metric for logic accelerators is throughput density, often listed in TOPS/mm^2, as it provides insight into the area efficiency of the in-memory computing architecture. While our peak experimental throughput density remains low, we estimate an impressive throughput density of 0.95 TOPS/mm^2 at a projected clock speed of 10 MHz due to the impressive integration density of even our standard crossbar array architecture. This improves to 6.10 TOPS/mm^2 and 12.4 TOPS/mm^2 for the alternative crossbar architectures due to their higher integration densities (smaller cell areas). Please note that the throughput density estimations given here are for array-level operations only and do not account for any peripheral circuitry or overhead; any consideration of such factors would naturally lead to a decrease in throughput density.*

A table comparing the current and predicted status of our work with other emerging logic accelerators for neural networks[10-23] is shown in **Supplementary Table 2**, with most results being adapted from ref. [10, 23]. From our work, we compare against the MNIST classification testing performed on a 64×10 subsection of the 64×32 array discussed in the main text, though we also include projections for operations on accelerators utilizing our alternative crossbar architectures (see **Supplementary Note 2-3**). The activation and weight resolution are both listed as 1-bit (ON/OFF) for our logic accelerators despite the analog nature of our NVMs due to binary images/weights being used for this proof-of-concept investigation. Further testing is needed to assess our ability to accurately and reproducibly assign multiple conductance states before/during logic operations. For all cases, clock speed is based on the time required for logic operations to be conducted (i.e., read operations when inputs are applied). For our accelerators, base refers to our typical read time of 100 ms (10 Hz), peak refers to our minimum confirmed read time of 4 µs (0.25 MHz), and projected refers to a theoretical clock speed as permitted by the final system; here, we use 10 MHz, the minimum clock speed reported for the contemporary works listed in the table, as our projected clock speed for the sake of comparison. The key metric for logic accelerators, throughput, in trillions of operations per second (TOPS), is estimated through the equation:

$$Throughput = \frac{Inputs(Rows \times Columns)}{Time}$$

Here, $Inputs$ refers to the number of inputs applied to the array, $Rows$ and $Columns$ refers to the number of rows and columns in the array being utilized, and $Time$ refers to the operational time ($^1/_f$). For assessing the throughput of our arrays, the number of columns being utilized at a time was restricted to 1 due to experimental limitations in assessing outputs in parallel; improvements in parallelization capabilities would therefore lead to a substantial improvement in throughput for all cases. In this manner, a base throughput of 1.23×10^{-7} TOPS, a peak throughput of 3.06×10^{-3} TOPS, and a projected (fully parallelized) throughput of 1.23 TOPS were estimated for our experimental 64×30 demonstration; this

increases to 1.31 TOPS for projected 64×32 accelerators based on the alternative architectures discussed in **Supplementary Note 2-3**. Another key metric for logic accelerators is throughput density, often listed in TOPS/mm^2, as it provides insight into the area efficiency of the in-memory computing architecture. While our peak experimental throughput density remains low, we estimate an impressive throughput density of 0.95 TOPS/mm^2 at a projected clock speed of 10 MHz due to the impressive integration density of even our primary crossbar array architecture, as shown in **Supplementary Table 1**. This improves to 6.10 TOPS/mm^2 and 12.4 TOPS/mm^2 for the designs discussed in **Supplementary Note 2-3**, respectively, due to their higher integration densities (smaller cell areas), indicating that our MoS_2-memtransistor-based crossbar array architectures have significant promise for dense, high throughput logic accelerators even in comparison to state-of-the-art technologies. Please note that the throughput density estimations given here are for array-level operations only and do not account for any peripheral circuitry or overhead; such factors would naturally lead to a decrease in throughput density but may in turn be offset by future developments in monolithically-integrated crossbar array architectures stacked directly on top of CMOS hardware[27-29].

Also note that estimations of throughput power efficiency (TOPS/W), a widely regarded metric for assessing neural network accelerator viability in the context of energy consumption, have been purposefully omitted from this benchmarking table to prevent any misleading comparisons of the power consumption of our work. As power consumption by peripherals can be an appreciable percentage of the entire power budget[10, 23], widespread consensus holds that a comparative analysis of power consumption between different accelerator architectures must account for peripherals to present an accurate perception of system efficiency. A discussion of power efficiency with this caveat in mind is presented below.

For the MNIST inference demonstration presented in the main manuscript, devices in the LRS state (logic “1”) are set to a conductance of ~50 nS. When an active pixel is fed to the array (V_{in} = 1 V), this

results in a dynamic energy consumption of 5 nJ/cell for a 100 ms read time (used for characterization and testing); this energy consumption can be as low as 0.2 pJ/cell for the experimentally confirmed minimum reliable read time of 4 μs presented in the Supplemental Information, which we consider the more relevant value for inference applications. Conversely, devices in the HRS state (logic "0") are set to a conductance of ~1 pS and dissipate comparatively negligible energy: approximately 0.1 pJ for a 100 ms read) and 4 aJ for a 4 μs read. Assuming an active device density of ~50% (960 LRS devices across a 64×30 sub-array), the total sub-array-level dynamic read energy is estimated at ~4.8 μJ under slow readout conditions and as low as ~192 pJ for fast readout, with an active power consumption of ~48 μW. Note that this estimation precludes contributions from sneak path current due to the tendency of binary weights to terminate sneak paths. Although a full TOPS/W metric requires knowledge of system-level power requirements including peripheral circuitry (DACs, ADCs, multiplexers, lookup tables, etc.)[10], these values allow us to conservatively estimate the energy efficiency of the array itself based on the throughput estimations provided above, translating to a power efficiency of ~638 TOPS/W for the crossbar array under peak conditions. It is extremely important to note that this high value represents a theoretical upper limit of efficiency and does not account for peripheral power draw. For example, previous investigations report that ADCs alone can account for 40–50% of the total power in similar systems[10]. We therefore expect that accounting for even minimal peripheral load will reduce the overall system-level efficiency by at least one or two orders of magnitude.

Supplementary Note 19

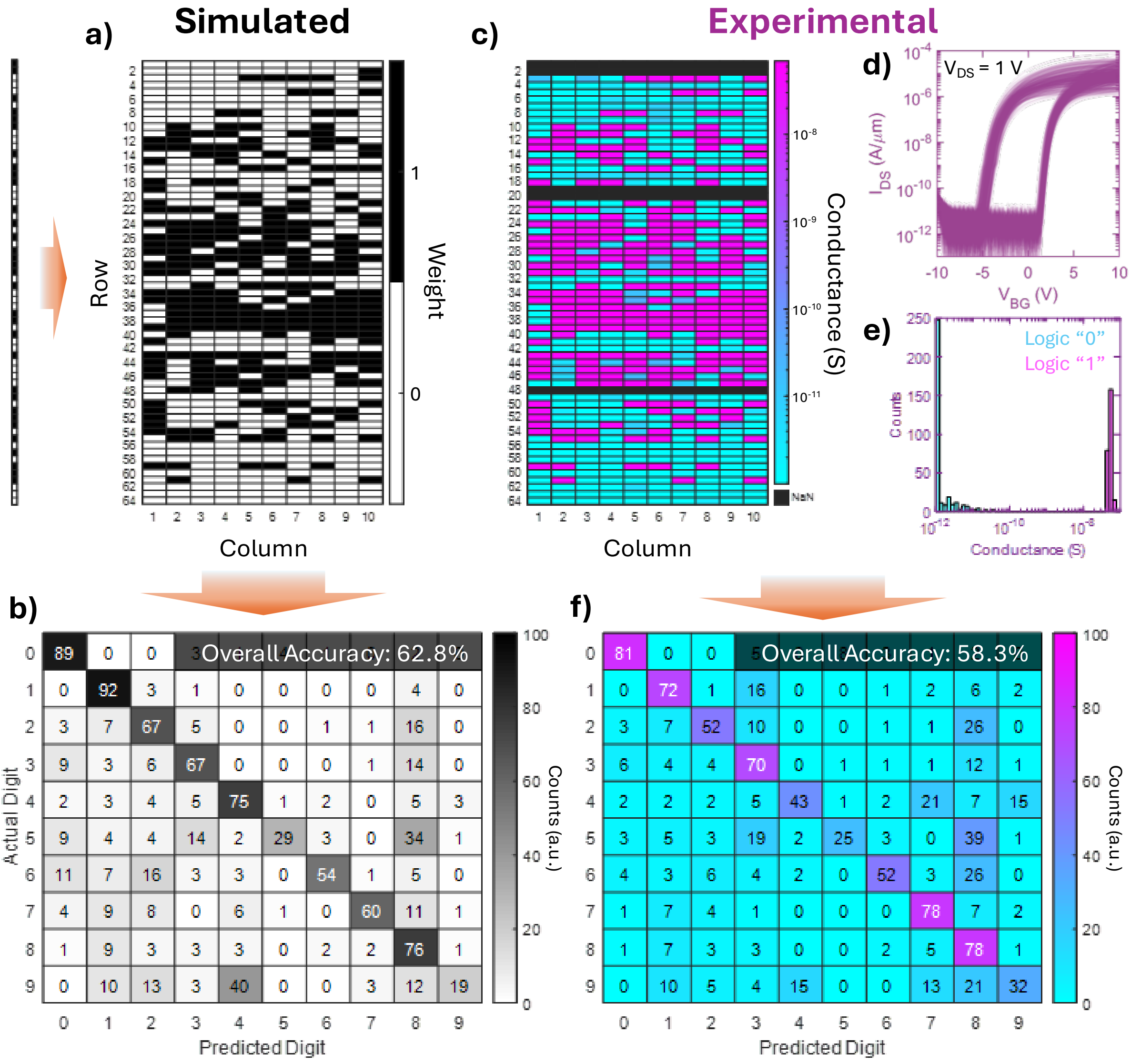

Supplementary Figure 17. Demonstration of 8×8-Pixel MNIST Handwritten Digit Classification. *The original MNIST images (28×28 pixels) were downscaled to 8×8 pixels and binarized to fit into a 64×10 sub-array for this demonstration. Downscaled images were then converted to 64×1 input vectors for input to the array. A dataset comprising 10,000 resized/reshaped images were then used for training and weight assignment; for this demonstration, simulated weights were split between logic "0" and "1" and later converted to targeted conductance states for hardware implementation. c) Heatmap showing the distribution of simulated weights following training. A test dataset of 1,000 resized/reshaped MNIST images was fed to the simulated array for verification of network inference/classification. d) Confusion matrix showing the classification results for the inference check described in (c). An overall accuracy of 62.8% was achieved. e) Heatmap showing the distribution of conductance states assigned to the hardware array in respect to the simulated weight distribution shown in (c), with weights of "1" mapped to a conductance state of ~50 nS and weights of "0" mapped to the OFF-state conductance (a few pS). Cells marked NaN either display an open circuit or high gate leakage; the overall yield of devices remained high at ~92.2%. f) Hysteresis characteristics of the 590 working devices in the 64×10 sub-array extracted at* V_{DS} *= 1 V, which show low intrinsic device-to-device variation, memory windows >5 V, and read margins >*10^4*. g) Histogram showing the distribution of the final conductance states (weights) represented in (e). h) Confusion matrix showing the classification results for hardware-based inference performed on the memtransistor array shown in (a) as per the conductance state (weight) distribution shown in (e). A test dataset comprising 1,000 resized/reshaped MNIST images was applied to the drain terminals of the array as voltage inputs (either 0 V or 1 V depending on the corresponding pixel value); output currents along corresponding columns/nodes were then individually registered and compared to all other outputs to determine the inferred digit for each case. An overall accuracy of 58.3% was registered.*

Supplementary References

1. Marega, G. M.; Ji, H. G.; Wang, Z.; Pasquale, G.; Tripathi, M.; Radenovic, A.; Kis, A., A large-scale integrated vector–matrix multiplication processor based on monolayer molybdenum disulfide memories. *Nature Electronics* **2023,** *6* (12), 991-998.
2. Feng, X.; Li, S.; Wong, S. L.; Tong, S.; Chen, L.; Zhang, P.; Wang, L.; Fong, X.; Chi, D.; Ang, K.-W., Self-Selective Multi-Terminal Memtransistor Crossbar Array for In-Memory Computing. *ACS Nano* **2021,** *15* (1), 1764-1774.
3. Lee, H.-S.; Sangwan, V. K.; Rojas, W. A. G.; Bergeron, H.; Jeong, H. Y.; Yuan, J.; Su, K.; Hersam, M. C., Dual-Gated MoS_2 Memtransistor Crossbar Array. *Advanced Functional Materials* **2020,** *30* (45).
4. Fu, S.; Park, J.-H.; Gao, H.; Zhang, T.; Ji, X.; Fu, T.; Sun, L.; Kong, J.; Yao, J., Two-Terminal MoS2 Memristor and the Homogeneous Integration with a MoS_2 Transistor for Neural Networks. *Nano Letters* **2023,** *23* (13), 5869-5876.
5. Kumar, P.; Zhu, K.; Gao, X.; Wang, S.-D.; Lanza, M.; Thakur, C. S., Hybrid architecture based on two-dimensional memristor crossbar array and CMOS integrated circuit for edge computing. *npj 2D Materials and Applications* **2022,** *6* (1).
6. Bala, A.; Sen, A.; Shim, J.; Gandla, S.; Kim, S., Back-End-of-Line Compatible Large-Area Molybdenum Disulfide Grown on Flexible Substrate: Enabling High-Performance Low-Power Memristor Applications. *ACS Nano* **2023,** *17* (14), 13784–13791.
7. Naqi, M.; Kang, M. S.; liu, N.; Kim, T.; Baek, S.; Bala, A.; Moon, C.; Park, J.; Kim, S., Multilevel artificial electronic synaptic device of direct grown robust MoS_2 based memristor array for in-memory deep neural network. *npj 2D Materials and Applications* **2022,** *6* (1).
8. Tang, B.; Veluri, H.; Li, Y.; Yu, Z. G.; Waqar, M.; Leong, J. F.; Sivan, M.; Zamburg, E.; Zhang, Y.-W.; Wang, J.; Thean, A. V.-Y., Wafer-scale solution-processed 2D material analog resistive memory array for memory-based computing. *Nature Communications* **2022,** *13* (1).
9. Chen, S.; Mahmoodi, M. R.; Shi, Y.; Mahata, C.; Yuan, B.; Liang, X.; Wen, C.; Hui, F.; Akinwande, D.; Strukov, D. B.; Lanza, M., Wafer-scale integration of two-dimensional materials in high-density memristive crossbar arrays for artificial neural networks. *Nature Electronics* **2020,** *3* (10), 638-645.
10. Xiao, T. P.; Bennett, C. H.; Feinberg, B.; Agarwal, S.; Marinella, M. J., Analog architectures for neural network acceleration based on non-volatile memory. *Applied Physics Reviews* **2020,** *7* (3).
11. Jouppi, N. P.; Young, C.; Patil, N.; Patterson, D.; Agrawal, G.; Bajwa, R.; Bates, S.; Bhatia, S.; Boden, N.; Borchers, A.; Boyle, R.; Cantin, P.-l.; Clifford Chao; Clark, C.; Coriell, J.; Daley, M.; Dau, M.; Dean, J.; Gelb, B.; Ghaemmaghami, T. V.; Gottipati, R.; Gulland, W.; Hagmann, R.; Ho, C. R.; Hogberg, D.; Hu, J.; Hundt, R.; Hurt, D.; Ibarz, J.; Jaffey, A.; Jaworski, A.; Kaplan, A.; Khaitan, H.; Killebrew, D.; Koch, A.; Kumar, N.; Lacy, S.; Laudon, J.; Law, J.; Le, D.; Leary, C.; Liu, Z.; Lucke, K.; Lundin, A.; MacKean, G.; Maggiore, A.; Mahony, M.; Miller, K.; Nagarajan, R.; Narayanaswami, R.; Ni, R.; Nix, K.; Norrie, T.; Omernick, M.; Penukonda, N.; Phelps, A.; Ross, J.; Ross, M.; Salek, A.; Samadiani, E.; Severn, C.; Sizikov, G.; Snelham, M.; Souter, J.; Steinberg, D.; Swing, A.; Tan, M.; Thorson, G.; Tian, B.; Toma, H.; Tuttle, E.; Vasudevan, V.; Walter, R.; Wang, W.; Wilcox, E.; Yoon, D. H. In *In-Datacenter Performance Analysis of a Tensor Processing Unit*, International Symposium on Computer Architecture, 2017.
12. Ltd., H. L. *Goya™ Inference Platform White Paper*; 2019; pp 1-14.
13. Chen, Y.; Luo, T.; Liu, S.; Zhang, S.; He, L.; Wang, J.; Li, L.; Chen, T.; Xu, Z.; Sun, N.; Temam, O., DaDianNao: A Machine-Learning Supercomputer. In *IEEE/ACM International Symposium on Microarchitecture*, IEEE: Cambridge, UK, 2014; pp 609-622.

14. Lee, J.; Kim, C.; Kang, S.; Shin, D.; Kim, S.; Yoo, H.-J., UNPU: An Energy-Efficient Deep Neural Network Accelerator With Fully Variable Weight Bit Precision. *IEEE Journal of Solid-State Circuits* **2018,** *54* (1), 173-185.
15. Bankman, D.; Yang, L.; Moons, B.; Verhelst, M.; Murmann, B., An Always-On 3.8 μJ/86% CIFAR-10 Mixed-Signal Binary CNN Processor With All Memory on Chip in 28-nm CMOS. *IEEE Journal of Solid-State Circuits* **2019,** *54* (1), 158-172.
16. Shafiee, A.; Nag, A.; Muralimanohar, N.; Balasubramonian, R.; Strachan, J. P.; Hu, M.; Williams, R. S.; Srikumar, V., ISAAC: A Convolutional Neural Network Accelerator with In-Situ Analog Arithmetic in Crossbars. *ACM SIGARCH Computer Architecture News* **2016,** *44* (3), 14-26.
17. Nag, A.; Balasubramonian, R.; Srikumar, V.; Walker, R.; Shafiee, A.; Strachan, J. P.; Muralimanohar, N., Newton: Gravitating Towards the Physical Limits of Crossbar Acceleration. *IEEE Micro* **2018,** *38* (5), 41-49.
18. Ankit, A.; Hajj, I. E.; Chalamalasetti, S. R.; Ndu, G.; Foltin, M.; Williams, R. S.; Faraboschi, P.; Hwu, W.-m.; Strachan, J. P.; Roy, K.; Milojicic, D. S. In *PUMA: A Programmable Ultra-efficient Memristor-based Accelerator for Machine Learning Inference*, International Conference on Architectural Support for Programming Languages and Operating Systems, 2019; pp 715-731.
19. Chi, P.; Li, S.; Xu, C.; Zhang, T.; Zhao, J.; Liu, Y.; Wang, Y.; Xie, Y., PRIME: a novel processing-in-memory architecture for neural network computation in ReRAM-based main memory. *ACM SIGARCH Computer Architecture News* **2016,** *44* (3), 27-39.
20. Bojnordi, M. N.; Ipek, E., Memristive Boltzmann machine: A hardware accelerator for combinatorial optimization and deep learning. In *IEEE International Symposium on High Performance Computer Architecture*, 2016; pp 1-13.
21. Bavandpour, M.; Sahay, S.; Mahmoodi, M. R.; Strukov, D. B., 3D-aCortex: an ultra-compact energy-efficient neurocomputing platform based on commercial 3D-NAND flash memories. *Neuromorphic Computing and Engineering* **2021,** *1* (1).
22. Jain, S.; Tsai, H.; Chen, C.-T.; Muralidhar, R.; Boybat, I.; Frank, M. M.; Wozniak, S.; Stanisavljevic, M.; Adusumilli, P.; Narayanan, P.; Hosokawa, K.; Ishii, M.; Kumar, A.; Narayanan, V.; Burr, G. W., A Heterogeneous and Programmable Compute-In-Memory Accelerator Architecture for Analog-AI Using Dense 2-D Mesh. *IEEE Transactions on Very Large Scale Integration (VLSI) Systems* **2023,** *31* (1), 114-127.
23. Aguirre, F.; Sebastian, A.; Gallo, M. L.; Song, W.; Wang, T.; Yang, J. J.; Lu, W.; Chang, M.-F.; Ielmini, D.; Yang, Y.; Mehonic, A.; Kenyon, A.; Villena, M. A.; Roldán, J. B.; Wu, Y.; Hsu, H.-H.; Raghavan, N.; Suñé, J.; Miranda, E.; Eltawil, A.; Setti, G.; Smagulova, K.; Salama, K. N.; Krestinskaya, O.; Yan, X.; Ang, K.-W.; Jain, S.; Li, S.; Alharbi, O.; Pazos, S.; Lanza, M., Hardware implementation of memristor-based artificial neural networks. *Nature Communications* **2024,** *15* (1).
24. Wan, Q.; Sharbati, M. T.; Erickson, J. R.; Du, Y.; Xiong, F., Emerging Artificial Synaptic Devices for Neuromorphic Computing. *Advanced Materials Technologies* **2019,** *4* (4).
25. Dai, S.; Zhao, Y.; Wang, Y.; Zhang, J.; Fang, L.; Jin, S.; Shao, Y.; Huang, J., Recent Advances in Transistor-Based Artificial Synapses. *Advanced Functional Materials* **2019,** *29* (42).
26. Chakraborty, I.; Ali, M.; Ankit, A.; Jain, S.; Roy, S.; Sridharan, S.; Agrawal, A.; Raghunathan, A.; Roy, K., Resistive Crossbars as Approximate Hardware Building Blocks for Machine Learning: Opportunities and Challenges. *Proceedings of the IEEE* **2020,** *108* (12), 2276-2310.
27. Jayachandran, D.; Pendurthi, R.; Sadaf, M. U. K.; Sakib, N. U.; Pannone, A.; Chen, C.; Han, Y.; Trainor, N.; Kumari, S.; McKnight, T. V.; Redwing, J. M.; Yang, Y.; Das, S., Three-dimensional integration of two-dimensional field-effect transistors. *Nature* **2024,** *625* (7994), 276-281.
28. Pendurthi, R.; Sakib, N. U.; Sadaf, M. U. K.; Zhang, Z.; Sun, Y.; Chen, C.; Jayachandran, D.; Oberoi, A.; Ghosh, S.; Kumari, S.; Stepanoff, S. P.; Somvanshi, D.; Yang, Y.; Redwing, J. M.; Wolfe, D.

E.; Das, S., Monolithic three-dimensional integration of complementary two-dimensional field-effect transistors. *Nature Nanotechnology* **2024,** *19* (7), 970-977.
29. Ghosh, S.; Zheng, Y.; Zhang, Z.; Sun, Y.; Schranghamer, T. F.; Sakib, N. U.; Oberoi, A.; Chen, C.; Redwing, J. M.; Yang, Y.; Das, S., Monolithic and heterogeneous three-dimensional integration of two-dimensional materials with high-density vias. *Nature Electronics* **2024,** *7* (892–903).
30. Prabhathan, P.; Sreekanth, K. V.; Teng, J.; Ko, J. H.; Yoo, Y. J.; Jeong, H.-H.; Lee, Y.; Zhang, S.; Cao, T.; Popescu, C.-C.; Mills, B.; Gu, T.; Fang, Z.; Chen, R.; Tong, H.; Wang, Y.; He, Q.; Lu, Y.; Liu, Z.; Yu, H.; Mandal, A.; Cui, Y.; Ansari, A. S.; Bhingardive, V.; Kang, M.; Lai, C. K.; Merklein, M.; Müller, M. J.; Song, Y. M.; Tian, Z.; Hu, J.; Losurdo, M.; Majumdar, A.; Miao, X.; Chen, X.; Gholipour, B.; Richardson, K. A.; Eggleton, B. J.; Wuttig, M.; Singh, R., Roadmap for phase change materials in photonics and beyond. *iScience* **2023,** *26* (10).
31. Aabrar, K. A.; Kirtania, S. G.; Fu-Xiang Liang; Gomez, J.; Jose, M. S.; Luo, Y.; Ye, H.; Dutta, S.; Ravikumar, P. G.; Ravindran, P. V.; Khan, A. I.; Yu, S.; Datta, S., BEOL-Compatible Superlattice FEFET Analog Synapse With Improved Linearity and Symmetry of Weight Update. *IEEE Transactions on Electron Devices* **2022,** *69* (4), 2094 - 2100.
32. Oberoi, A.; Dodda, A.; Liu, H.; Terrones, M.; Das, S., Secure Electronics Enabled by Atomically Thin and Photosensitive Two-Dimensional Memtransistors. *ACS Nano* **2021,** *15* (12), 19815-19827.
33. Dodda, A.; Trainor, N.; Redwing, J. M.; Das, S., All-in-One, Bio-Inspired, and Low-Power Crypto Engines for Near-Sensor Security Based on Two-Dimensional Memtransistors. *Nature Communications* **2022,** *13* (1), 1-12.
34. Zheng, Y.; Ravichandran, H.; Schranghamer, T. F.; Trainor, N.; Redwing, J. M.; Das, S., Hardware Implementation of Bayesian Network Based on Two-Dimensional Memtransistors. *Nature Communications* **2022,** *13* (1), 1-11.
35. Deng, W.; Yan, X.; Wang, L.; Yu, N.; Luo, W.; Mai, L., Two-dimensional materials based memtransistors: Integration strategies, switching mechanisms and advanced characterizations. *Nano Energy* **2024,** *128*.
36. Zhang, S.; Liu, Y.; Zhou, J.; Ma, M.; Gao, A.; Zheng, B.; Li, L.; Su, X.; Han, G.; Zhang, J.; Shi, Y.; Wang, X.; Hao, Y., Low Voltage Operating 2D MoS_2 Ferroelectric Memory Transistor with $Hf_{1-x}Zr_xO_2$ Gate Structure. *Nanoscale Research Letters* **2020,** *15* (1), 1-9.
37. Kim, I.-J.; Lee, J.-S., Ferroelectric Transistors for Memory and Neuromorphic Device Applications. *Advanced Materials* **2022,** *35* (22).
38. Tan, C.; Wu, H.; Zhao, M.; Jili, X.; Yang, L.; Gao, L.; Wang, Z., Gate-Switchable BST Ferroelectric MoS_2 FETs for Non-Volatile Digital Memory and Analog Memristor. *Advanced Functional Materials* **2024,** *34* (39).
39. Larcher, L.; Padovani, A., undamental reliability issues of advanced charge-trapping Flash memory devices. In *IEEE International Conference on Electronics, Circuits and Systems*, IEEE: Athens, Greece, 2010.
40. Spassov, D.; Paskaleva, A., Challenges to Optimize Charge Trapping Non-Volatile Flash Memory Cells: A Case Study of HfO_2/Al_2O_3 Nanolaminated Stacks. *Nanomaterials* **2023,** *13* (17).
41. Paskaleva, A.; Rommel, M.; Hutzler, A.; Spassov, D.; Bauer, A. J., Tailoring the Electrical Properties of HfO_2 MOS-Devices by Aluminum Doping. *ACS Applied Materials & Interfaces* **2015,** *7* (31), 17032–17043.
42. Spassov, D.; Paskaleva, A.; Krajewski, T. A.; Guziewicz, E.; Luka, G., Hole and electron trapping in HfO_2/Al_2O_3 nanolaminated stacks for emerging non-volatile flash memories. *Nanotechnology* **2018,** *29*.
